# In situ atomic-scale observation of oxygen-driven core-shell formation in Pt_3_Co nanoparticles

**DOI:** 10.1038/s41467-017-00161-y

**Published:** 2017-08-07

**Authors:** Sheng Dai, Yuan You, Shuyi Zhang, Wei Cai, Mingjie Xu, Lin Xie, Ruqian Wu, George W. Graham, Xiaoqing Pan

**Affiliations:** 1 Department of Chemical Engineering and Materials Science, University of California Irvine, Irvine, CA 92697 USA; 2 Department of Physics and Astronomy, University of California Irvine, Irvine, CA 92697 USA; 3 School of Physics and Electronics, Yancheng Teachers University, Yancheng, Jiangsu 224002 China; 4 Department of Materials Science and Engineering, University of Michigan, Ann Arbor, MI 48109 USA; 5 National Laboratory of Solid State Microstructures and College of Engineering and Applied Sciences, Nanjing University, Nanjing, Jiangsu 210094 P.R. China

## Abstract

The catalytic performance of core-shell platinum alloy nanoparticles is typically superior to that of pure platinum nanoparticles for the oxygen reduction reaction in fuel cell cathodes. Thorough understanding of core-shell formation is critical for atomic-scale design and control of the platinum shell, which is known to be the structural feature responsible for the enhancement. Here we reveal details of a counter-intuitive core-shell formation process in platinum-cobalt nanoparticles at elevated temperature under oxygen at atmospheric pressure, by using advanced in situ electron microscopy. Initial segregation of a thin platinum, rather than cobalt oxide, surface layer occurs concurrently with ordering of the intermetallic core, followed by the layer-by-layer growth of a platinum shell via Ostwald ripening during the oxygen annealing treatment. Calculations based on density functional theory demonstrate that this process follows an energetically favourable path. These findings are expected to be useful for the future design of structured platinum alloy nanocatalysts.

## Abstract (web summary)

Core-shell platinum alloy nanoparticles are promising catalysts for oxygen reduction, however a deeper understanding of core-shell formation is still required. Here the authors report oxygen-driven formation of core-shell Pt_3_Co nanoparticles, seen at the atomic scale with in situ electron microscopy at ambient pressure.

## Introduction

Core-shell structured platinum-metal (Pt-M, M=Fe, Co, Ni, etc.) nanoparticles (NPs) are presently attracting considerable research attention since they are a promising alternative to traditional Pt NPs as the oxygen reduction reaction (ORR) catalyst in polymer electrolyte membrane fuel cells^1–7^. Not only does the core-shell Pt-M catalyst reduce the cost and demand of precious metals, but it also shows as great as a 22× enhanced ORR activity relative to the pure Pt catalyst^8^. Since the Pt shell (on the bimetallic core), with its compressive strain and ligand effect, has been identified as the structural feature responsible for enhancing the ORR activity^8–14^, post-synthesis treatments have been investigated to control the Pt shell formation in order to obtain further catalytic improvement. For instance, acid leaching^8–10^ or high-temperature annealing^11–13^ have long been employed to enrich the near-surface region of the Pt-M catalyst with Pt.

Although these prior studies have demonstrated the ability to generate a Pt shell on the bimetallic core, it still remains a considerable challenge to precisely control the uniformity and thickness of the Pt skin at the atomic scale. Guided largely by theoretical simulation results^14–16^, trial-and-error approaches are currently used to explore treatment conditions, while the structural evolution of the Pt shell can only be speculated upon, according to post-treatment characterization. As a result, some favorable structures at an intermediate state can be easily missed, and the whole process of core-shell formation is still unclear. Thus, crucial questions relating to core-shell formation exist as follows: (1) Can the whole Pt-M core-shell formation process be directly followed at the atomic scale in real time? (2) What drives the core-shell formation under the processing conditions? (3) Are there possibly new mechanisms that can be utilized to grow and tailor the Pt shell? To provide answers to these significant questions, here we investigate the core-shell formation process in Pt_3_Co NPs, by using our advanced in situ transmission electron microscopy technique at atmospheric gas pressure^17, 18^. An unexpected oxygen-driven core-shell formation process is observed at the atomic scale, and the corresponding mechanism is proposed based on our density functional theory (DFT) calculations. These findings should help pave the way for the surface engineering and atomic-scale control of improved Pt-M core-shell nanocatalysts.

## Results

### Sample information

**Fig. 1:**
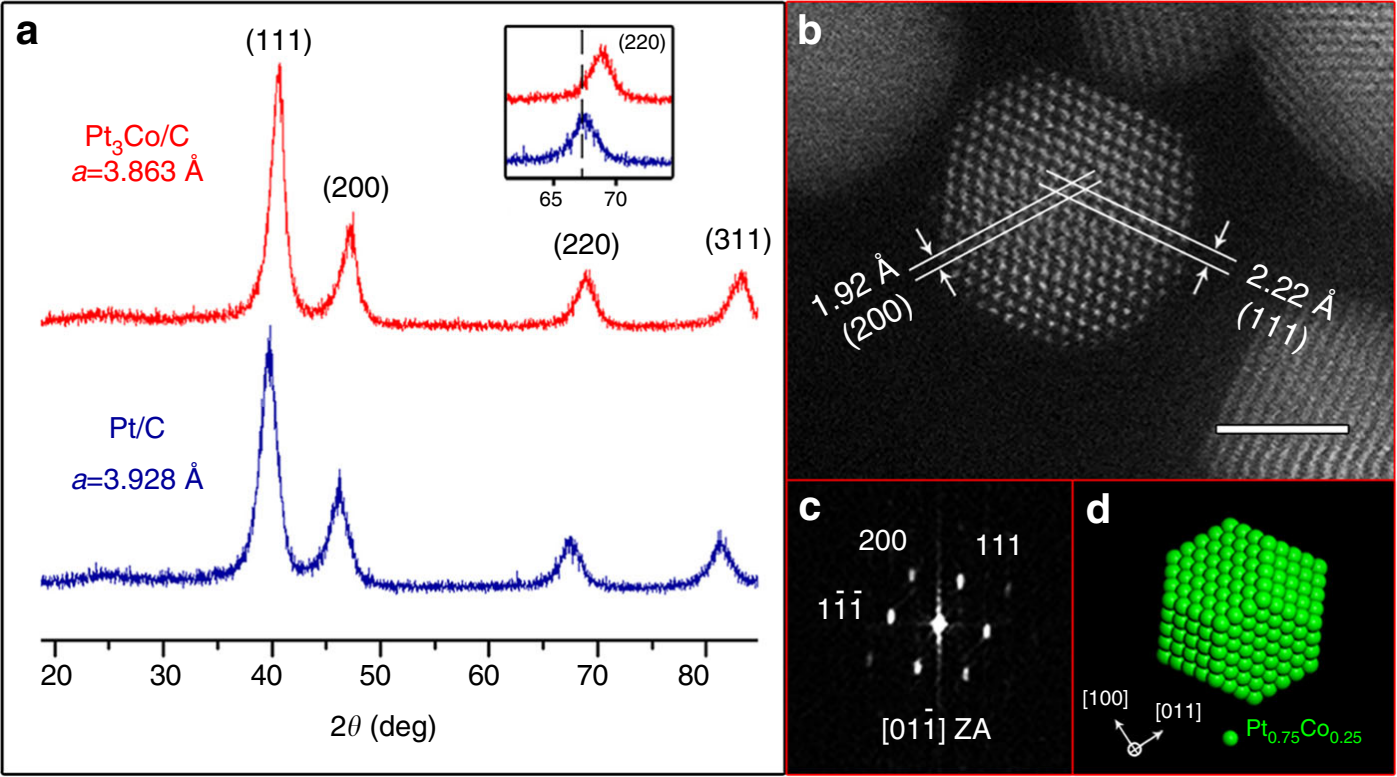
XRD and STEM characterization results of the Pt_3_Co/C sample. **a** X-ray diffraction patterns of Pt_3_Co/C and Pt/C samples. The inset is an enlarged region of the (220) diffraction peaks of both samples. **b** HAADF-STEM image of a Pt_3_Co NP along the $[01\mathop {1}\limits^ - ]$ zone axis. Scale bar, 2 nm. **c** FFT pattern of the NP in **b**, illustrating the disordered fcc structure with an absence of (100) superlattice spots. **d** Projection of a truncated octahedron model along the <110> zone axis. Green spheres correspond to randomly distributed Pt and Co atoms in the disordered Pt_3_Co phase

A carbon-supported Pt_3_Co powder catalyst (Pt_3_Co/C), provided by Tanaka Kikinzoku Kogyo (TKK) Co. Ltd., was used for our study, and a carbon-supported pure Pt (Pt/C) catalyst, also from TKK, was used as a reference. The chemical composition of the Pt_3_Co/C sample was confirmed by energy dispersive X-ray spectroscopy (EDS) as the atomic ratio of Pt to Co is 3:1 (Supplementary Fig. 1). Figure 1a presents X-ray diffraction (XRD) results from the Pt_3_Co/C and Pt/C samples. The four diffraction peaks corresponding to (111), (200), (220) and (311) planes of the face-centred cubic (fcc) structure are evident in the patterns of both samples, while the broad peak at around 25˚ is attributed to the carbon support^12, 19^. Significantly, (100) and (110) peaks, characteristic of the ordered primitive cubic (L1_2_) Pt_3_Co phase^20^, are not apparent in the pattern of our Pt_3_Co/C sample. Hence, the NPs are present in the disordered Pt_3_Co phase at this stage of the study. Meanwhile, it is obvious that the peak positions of the Pt_3_Co/C sample (red line) are shifted to higher angles (see the inset of Fig. 1a), reflecting the concomitant lattice contraction due to the relatively smaller Co atoms incorporated into the Pt structure. The calculated lattice parameters, ${a_{{\rm{P}}{{\rm{t}}_{\rm{3}}}{\rm{Co}}}} = 3.863$Å and ${a_{{\rm{Pt}}}} = 3.928$Å, are consistent with the reported lattice parameter range of Pt_3_Co and Pt^12, 21^, confirming the compositions of our samples.

Aberration-corrected scanning transmission electron microscopy (AC-STEM) was utilized for detailed characterization of the Pt_3_Co NPs. Figure 1b is a typical high-angle annular dark field (HAADF) image of the Pt_3_Co NPs at this stage of the study. The single-crystallinity of the fcc structure is reflected in the atomic-scale image, and the corresponding fast Fourier transform (FFT) shows the absence of (100) superlattice spots, confirming that the Pt_3_Co NPs are in the disordered phase. In addition, electron microscopy characterization also shows the morphology of these NPs. Based on surface energy calculations^22^, the equilibrium shape of Pt-Co NPs is expected to be a truncated octahedron consisting predominantly of {100} and {111} facets. Apparently, the STEM image along the <110> zone axis (Fig. 1b) is consistent with the projection of the truncated octahedron model along the same direction, as shown in Fig. 1d.

### In situ oxygen annealing experiment

To explore the core-shell formation process, in situ experiments were performed in a Protochips Atmosphere gas cell system (see schematic in Supplementary Fig. 2), which allows for dynamic observation of nanomaterials heated under elevated gas pressure inside the transmission electron microscope. Here we carried out the in situ high temperature annealing in pure oxygen (purity 99.9995%) at pressures as high as one atmosphere (760 Torr), which has not been done in previous in situ TEM studies regarding the Pt-M core-shell formation^23, 24^. The oxygen annealing was performed at 720 °C, after heating from room temperature at a very high rate of 5 °C/s.

**Fig. 2:**
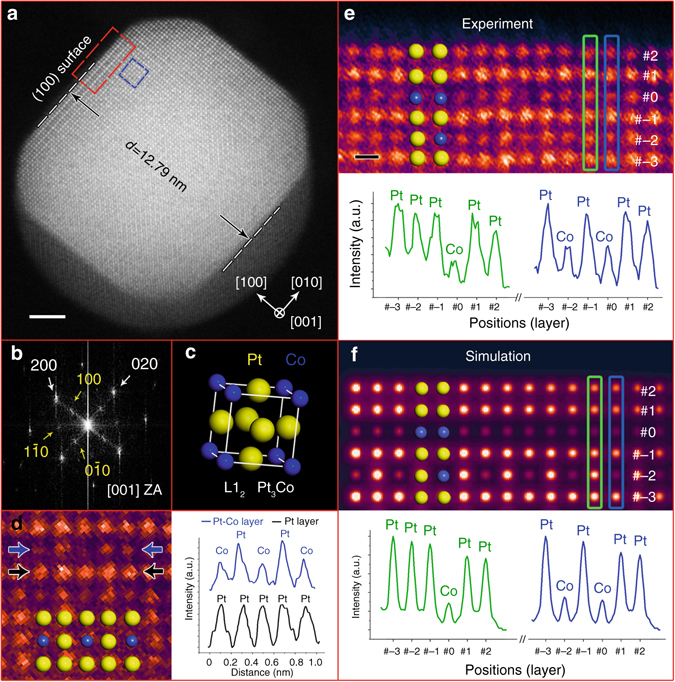
In situ results from a Pt_3_Co NP after oxygen annealing at 720 °C. **a** HAADF-STEM image taken inside the gas cell showing the Pt_3_Co NP after a 30-min annealing in oxygen atmosphere. Scale bar, 2 nm. **b** FFT pattern of the NP in **a**. **c** Unit cell of the L1_2_ phase illustrating the ordered intermetallic Pt_3_Co structure. *Yellow* and *blue spheres* represent Pt and Co atoms, respectively. **d** A false-colour cropped image of the ordered intermetallic feature taken from the *blue box* in **a** and the intensity profiles taken along the lines indicated by the *blue* and *black arrows*, respectively. **e** Enlarged false colour image of the (100) surface taken from the *red box* in **a**, and the intensity profiles taken along the atomic layers marked by *green* and *blue rectangles* showing the segregated Pt-rich surface. Scale bar, 2 Å. **f** Simulated HAADF-STEM image of a L1_2_ intermetallic Pt_3_Co model with Pt-segregated surfaces, and the intensity profiles from the two atomic layers marked by *green* and *blue rectangles*

Figure 2a shows a Pt_3_Co NP (viewed along the [001] zone axis) after annealing in oxygen for 30 min. It can be seen that the particle still has a truncated octahedron shape, consistent with the projection model in Supplementary Fig. 3. As illustrated in Fig. 2a, the diameter (*d*) across two opposite (100) surfaces is 12.79 nm. In the corresponding FFT pattern, it is clear that both the (100) and (110) superlattice spots (as marked by yellow arrows in Fig. 2b) can be observed at this time. The appearance of these superlattice features, representative of the ordered L1_2_ phase, which were not seen in the original sample, indicates that a disorder-to-order phase transition^25, 26^ has occurred in this Pt_3_Co NP during the oxygen annealing process. Meanwhile, the presence of the L1_2_ structure can also be identified from the atomic-resolution STEM image (Fig. 2d). Due to the Z contrast in HAADF-STEM images^27^, the Pt columns will have a higher intensity than the Co columns. Therefore, along the [001] zone axis, the L1_2_ unit cell can be revealed as a Pt-Co mixed (200) layer next to a pure Pt (200) layer. As shown in Fig. 2d, the intensity profiles taken along the lines indicated by the blue and black arrows clearly demonstrate that this is the case. This situation is markedly different from that in the disordered alloy phase, which shows an equal intensity in all columns because of the randomly distributed Pt and Co atoms.

It should also be noted that Pt surface segregation was taking place during the disorder-to-order transition in the Pt_3_Co NPs. Figure 2e shows the enlarged false colour image of the (100) surface (indicated by the red box in Fig. 2a). The third layer from the top surface is defined as layer #0 and other layers are labelled in sequence. Here, the intensity profile in Fig. 2e taken from two adjacent (020) planes illustrates that the atomic columns from either layer #1 or #2 have a comparable intensity to that of the inner Pt columns. In contrast, the columns from layer #0 exhibit a similar intensity to the inner Co columns. To confirm this segregated surface configuration, image simulations were performed utilizing the multi-slice method^28^. The atomic model was established as close to the observed particle as possible by considering the intermetallic structure, the geometry of the truncated octahedron, and the segregated Pt-Pt-Co surface (more details are provided in Supplementary Note 1). Figure 2f displays the simulated output, and the corresponding intensity profiles show a qualitative agreement with our experimental result. Therefore, we confirm the Pt-Pt-Co surface configuration, indicating that Pt surface segregation was taking place during oxygen annealing, i.e., that Pt atoms are driven to the surface (layer #1 and #2) and Co atoms are correspondingly displaced inside, leaving a Co-rich layer (#0) below the top surface (more details confirming the Pt surface segregation on (111) surfaces are provided in Supplementary Figs. 4 and 7).

**Fig. 3:**
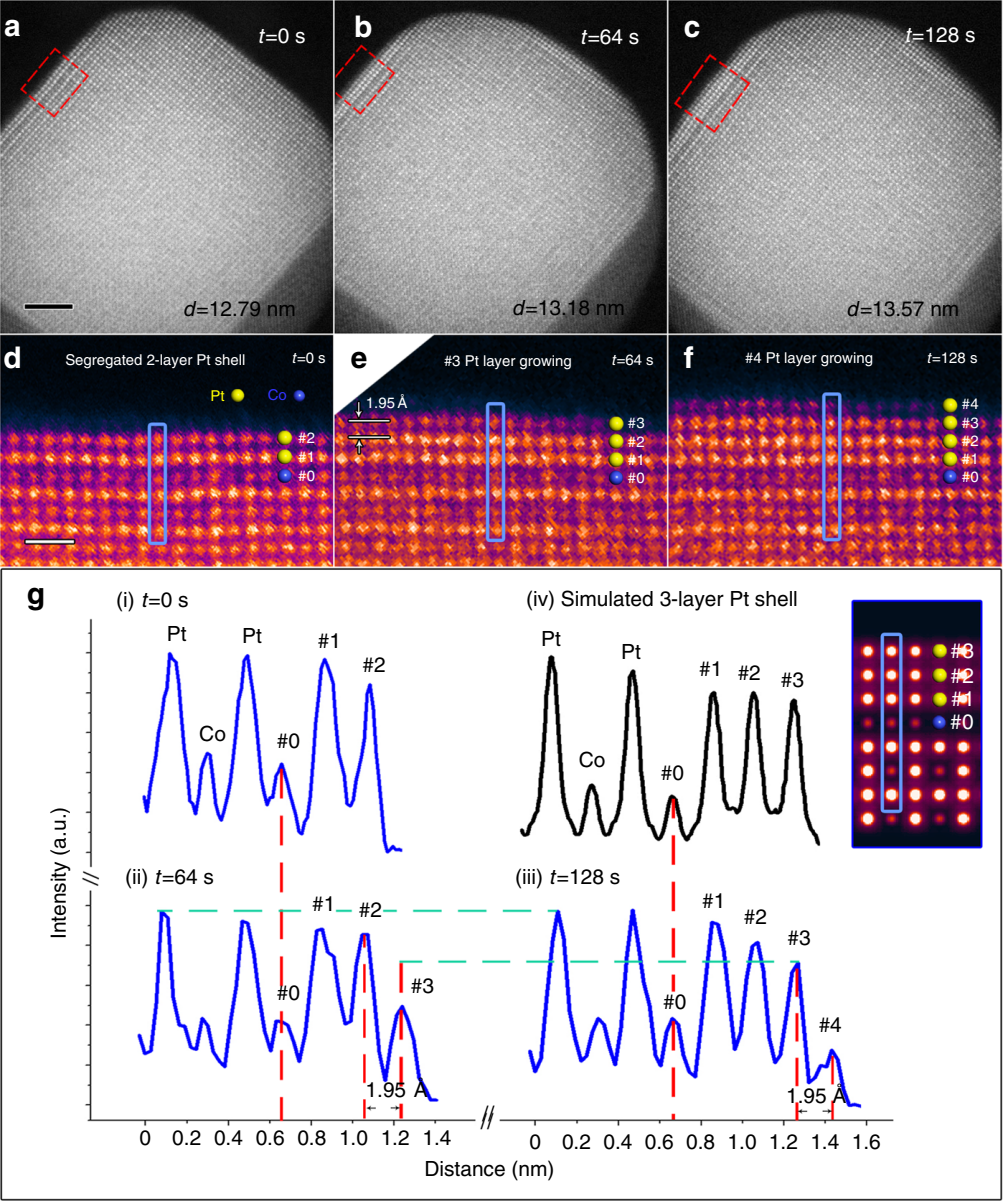
Layer-by-layer growth of the (100) Pt shell during oxygen annealing. **a**–**c** Sequential HAADF images taken at 0, 64, and 128 s during oxygen annealing at 300 °C, respectively. Scale bar, 2 nm. **d**–**f** Corresponding enlarged false-colour panels of the (100) surface (indicated by red boxes) in **a**–**c**, respectively. Yellow and blue spheres represent Pt and Co atoms, respectively. Scale bar, 5 Å. **g** Intensity profiles taken along the blue lines in **d**–**f**, and the simulation result, respectively. The inset shows the simulated image of the three-layer Pt shell on the (100) surface

After 30-min of oxygen annealing at 720 °C, the temperature was lowered to 300 °C, at a cooling rate of 1 °C/s, while the oxygen pressure was maintained at 760 Torr. Figure 3a, taken at the moment when the temperature just reached 300 °C, is labeled as *t* = 0 s for that time. The diameter, *d*_0s_, of the given NP at this time was still 12.79 nm, the same as the diameter measured at 720 °C. The enlarged surface configuration (Fig. 3d), displaying the 2-layer Pt-rich shell, proves its stability during the cooling process.

Sequential high-resolution HAADF images were then taken at a scanning speed at one frame every 16 s at 300 °C. As shown in Fig. 3b, the Pt_3_Co NP was found to be larger at an elapsed time of 64 s. At this stage, the measured diameter, *d*_64s_, was 13.18 nm, showing an increase of Δ*d* = 3.90 Å. In the corresponding enlarged panel (Fig. 3e), it is evident that an extra atomic layer (layer #3) has grown on the (100) surface. The lattice spacing between layer #2 and layer #3 is found to be 1.95 Å, which is exactly half of Δ*d*. This indicates another extra atomic layer has also started growing on the opposite (100) facet at the lower right corner of Fig. 3b. Similarly, by comparing Figs. 3a and 3b, an extra layer can also be identified on the other two (100) surfaces (the one at the top right and the one at lower left).

Furthermore, at an elapsed time of 128 s (Fig. 3c), the measured diameter, *d*_128s_ = 13.57 nm, shows a further increase of 3.90 Å, demonstrating that the particle continued to undergo layer-by-layer growth at (100) surfaces. The high-resolution image (Fig. 3f) also illustrates the newly grown layer #4. Finally, this Pt_3_Co particle reached a limit after finished the growth of layer #5, which then remained stable at 300 °C (see Supplementary Fig. 5 and Supplementary Note 2). Meanwhile, it can be confirmed that the layer-by-layer growth only happens at (100) but not (111) surfaces by further analysis of the sequential HAADF images (see Supplementary Fig. 6 and Supplementary Note 3).

To determine the composition of the newly grown layers, intensity profiles are obtained from the same (020) plane (indicated by blue boxes in Figs. 3d–f) at *t* = 0, 64, and 128 s, respectively. As shown in Fig. 3g, the new layers #3 and #4 can be quantitatively represented in the sequential intensity profiles. By comparing profiles (ii) and (iii), it can be found that the intensity of layer #3 is increasing during the interval of 64-128 s. This evolution appears to reflect growth of the surface (i.e., growth parallel to the (001) zone axis of the NP). For example, at time 64 s, layer #3 was in its primary stage of growth,with only a few atoms attached at this layer, so that a relatively weak intensity was exhibited, as shown in the profile (ii). But thereafter, the intensity became stronger as more and more atoms were attached until the growth was finished in layer #3 and started in layer #4 (see profile (iii)).

In order to determine the composition of newly grown layer #3, an intensity analysis may be carried out using the profile (iii), in which layer #3 is considered as completely grown. It is obvious that the columns in layer #3 exhibit a similar intensity to that of the segregated Pt layers #1 and #2, which is higher than that of the inner Co columns. For confirmation, an image simulation was performed based on a segregated intermetallic Pt_3_Co model with a three-layer pure Pt shell. According to the simulation results, the intensity profile of the three-layer Pt shell shows the same trend as the corresponding layers #1-#3 in the experimental profile (iii), demonstrating the newly grown layer we observed is, indeed, a Pt-rich shell. Similarly, the following new layers (#4 and #5) were also found to be Pt-rich.

To summarize, we have found that, upon oxygen annealing, an initially disordered Pt_3_Co NP transforms into an ordered intermetallic Pt_3_Co NP with a thin Pt shell within 30 min at 720 °C, and that additional surface layers of Pt may be added to the resulting NP by further annealing at 300 °C. This end result was also confirmed by HAADF-STEM and X-ray photoelectron spectroscopy (XPS) measurements performed on Pt_3_Co samples that were treated by a similar ex situ oxygen annealing treatment (see Supplementary Table 1, Supplementary Figs. 7–9 and Supplementary Notes 4–5). Such ordered core-shell NPs might possess both excellent electrocatalytic activity and stability for the ORR according to the prior studies^12^.

Traditionally, core-shell-structured Pt-M catalysts were made by a reductive annealing or a vacuum annealing treatment, according to either in situ^23, 24^ or ex situ^11, 12, 29–31^ observation. Among the existing reports, oxygen annealing has not been considered as a post treatment to enrich the near-surface region of the Pt-M catalyst with Pt, since it is expected to induce the segregation of the less-noble M oxides (e.g., CoO, NiO) on the NP surface^32–34^, blocking Pt atoms from participating in electrocatalytic reactions. However, under the conditions of our in situ experiment, as described above, no Co oxidation was observed. We believe this “anti-oxidation” phenomenon may be attributed to the ordered intermetallic Pt_3_Co structure. To prove our hypothesis, another in situ experiment was performed for comparison. This time, the original Pt_3_Co sample was annealed in oxygen at 300–500 °C for 30 min. Under such a treatment condition, the disorder-to-order transition did not occur; yet, high resolution HAADF images clearly revealed a CoO layer on the surface (see Supplementary Fig. 10), showing that disordered Pt_3_Co NPs may be readily oxidized in the oxygen environment. This oxidation behavior is consistent with previous in situ TEM studies showing Co segregation and oxidation in Pt-Co NPs that were in the disordered alloy phase^32^. Therefore, the difference between disordered and ordered structure is, indeed, responsible for dissimilar oxidation behavior of the Pt-M NPs. Rapid heating to 720 °C (e.g., at a rate of 5 °C/s) is also necessary in order to limit the opportunity for oxidation in the intermediate temperature range of 300–500 °C before the disorder-to-order transition can be triggered at the temperature of 720 °C, which allows Pt surface segregation to take place instead of the formation of cobalt oxide during this stage.

### DFT calculations

To better understand the mechanism of the oxygen-induced core-shell formation, first principle calculations based on density functional theory were performed. Several possible (100), (110), and (111) surfaces of ordered intermetallic Pt_3_Co were investigated with a periodic slab model that has either (1 × 1) or (2 × 1) unit cell in the lateral plane (Supplementary Fig. 11). The stability of each normal surface can be quantitatively described by its surface formation energy (*E*_f_) that is defined as

1 $${E_{\rm{f}}} = {E_{{\rm{slab}}}} - \left( {{m_{{\rm{Pt}}}} - {\rm{3}}{n_{{\rm{Co}}}}} \right){\mu _{{\rm{Pt}}}} - {n_{{\rm{Co}}}}{E_{{\rm{P}}{{\rm{t}}_3}{\rm{Co}}}}$$

where *E*_slab_ is the total energy of the slab, and *m*_Pt_ and *n*_Co_ represent the numbers of Pt and Co atoms in each case, respectively. *μ*_Pt_ is the chemical potential of a Pt atom and is used as the variable. ${E_{{\rm{P}}{{\rm{t}}_3}{\rm{Co}}}}$ is the formation energy of one Pt_3_Co unit in the ordered L1_2_ phase.

**Fig. 4:**
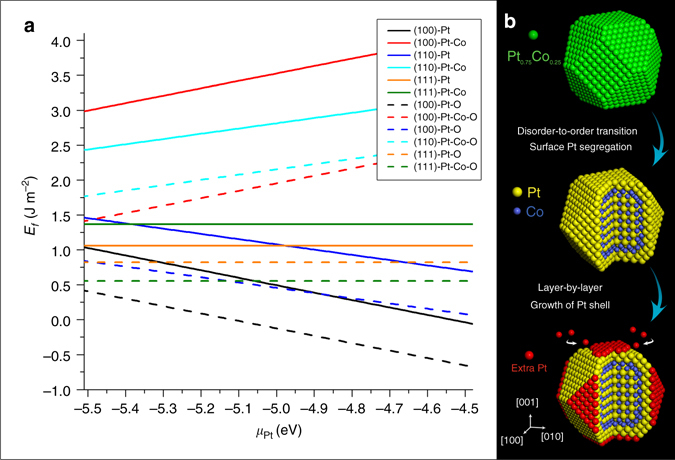
Structure evolution during oxygen-induced core-shell formation. **a** DFT calculation results of the formation energy on several possible surfaces of the ordered intermetallic Pt_3_Co phase. **b** Schematic diagrams showing the process of the oxygen-driven core-shell formation in Pt_3_Co NPs

The calculated formation energies of all possible surfaces (coloured solid lines) are plotted as a function of the chemical potential *μ*_Pt_ in Fig. 4a. In our experiment, the chemical potential *μ*_Pt_ should be in the range from −5.5 eV (Pt atoms from bulk form) to −4.5 eV (Pt atoms from (100) surfaces). It is clear that every pure Pt surface has lower energy than the corresponding Pt-Co intermixed surface, indicating the pure Pt surface is more energetically favorable in the ordered intermetallic Pt_3_Co.

For considering the effect of an oxygen environment, the surface is then simulated by a slab with oxygen adatoms. The surface formation energy of an oxygen-covered (O-covered) surface can be determined according to

2 $${E_{\rm{f}}} = {E_{{\rm{slab}}}} - \left( {{m_{{\rm{Pt}}}} - {\rm{3}}{n_{{\rm{Co}}}}} \right){\mu _{{\rm{Pt}}}} - {n_{{\rm{Co}}}}{E_{{\rm{P}}{{\rm{t}}_3}{\rm{Co}}}} - \frac{1}{2}{E_{{{\rm{O}}_2}}}$$

where ${E_{{{\rm{O}}_2}}}$ is the total energy of oxygen.

In Fig. 4a, the colored dashed lines represent the formation energies of the surfaces fully covered with oxygen atoms. It can be found that every dashed line is below the same-colored solid line in the given range of *μ*_Pt_, indicating the oxygen environment can significantly reduce the formation energy of every surface. Although the formation energies of Pt-Co mixed surfaces drop more than those of pure Pt surfaces, the (100) facet with pure Pt in the topmost layer is still the most favorable configuration. One may note that the (111)-Pt-Co-O surface has comparable formation energy to the (100)-Pt-O surface at the Pt-poor end. However, the segregation of Co from the bulk alloy toward the (111) surface is effectively frustrated even in an oxidation environment due to the high energy cost of breaking the Pt-Co bonds (anti-segregation energy), according to the investigations of Balbuena et al.^16^. Therefore, the DFT data here and also in references^16, 35^ indicate that surface oxidation of Co cannot happen on the ordered core-shell Pt_3_Co nanoparticles. The Pt shell on the ordered intermetallic core can thus hinder Co segregation and protect the NPs from surface oxidation^36^.

## Discussion

Then, we discuss a possible mechanism for the extra layer-by-layer growth of the Pt-shell. According to the DFT simulation, the Pt (100) surface exhibits the lowest formation energy of all calculated surfaces while taking the oxygen environment into account. Notably, if *μ*_Pt_ is higher than −5.0 eV (in the scenarios that Pt atoms are taken away from (100), (110), or (111) surfaces), the formation energy of the O-covered Pt (100) surface (black dashed line in Fig. 4a) is the only one to become negative. This suggests the (100) surface tends to grow by attracting atoms from other Pt sources, such as isolated Pt atoms or clusters, which are commonly found during the high temperature annealing process of Pt-M NPs^10^. Based on previous research^37^, Pt atoms diffusing along surfaces, or possibly gas-phase PtO_2_ molecules, possess a high mobility under oxidizing conditions, and Pt is thus expected to easily migrate and attach on to larger NPs during an oxygen annealing treatment, essentially undergoing Ostwald ripening^38^. This possibility is supported by our in situ STEM images in Supplementary Fig. 12, which reveal the high mobility of Pt atoms/clusters in an oxygen environment. Such a scenario is also consistent with the modest change in the size distribution (Supplementary Table 2 and Supplementary Fig. 13) of our Pt_3_Co NPs: NPs with diameters less than 4 nm tend to disappear during the oxygen annealing treatment. Of course, the diffusion of Pt atoms can take place over a wide temperature range^37^, but we found the layer-by-layer growth is relatively easy to observe, and the ordered intermetallic structure can be maintained at the temperature of 300 °C.

We believe that oxygen-driven Pt shell formation may provide a new avenue for the structural engineering of the Pt shell in Pt-M NP catalysts. Among the traditional reductive annealing^11, 12^ and vacuum annealing treatments^27, 28^, the formation of a Pt shell only involves surface segregation. As a result, the Pt shell is usually <3 atomic layers thick, since the displacive behavior only takes place at the very near-surface region. However, the Pt shell formation under oxygen annealing may further exploit Ostwald ripening, allowing a thicker Pt shell (e.g., up to five layers of Pt in our in situ experiment) to be achieved, which may provide more opportunities to optimize the catalytic stability^39^ and ORR activity^40^.

In conclusion, by performing the in situ TEM study under atmospheric pressures, we have observed an oxygen-driven core-shell formation process in a Pt_3_Co fuel-cell-cathode catalyst. The whole process takes place in two steps according to our in situ experiment and DFT calculations (Fig. 4b). Firstly, the phase transition from disordered Pt_3_Co alloy to ordered L1_2_ intermetallic Pt_3_Co happens while the NPs are annealing in an oxygen atmosphere at 720 °C. In this step, an initial two-layer Pt-rich shell is formed through surface segregation, which effectively blocks Co oxidation. Secondly, when the temperature is lowered to 300 °C, Pt atoms from other clusters migrate and attach onto the (100) Pt surface of the ordered Pt_3_Co NPs, resulting in further layer-by-layer growth of the Pt shell in an oxygen environment. Our work not only demonstrates the stability of the ordered intermetallic core-shell Pt_3_Co catalyst in an oxidizing environment, but it also shows the advantage of the oxygen annealing in synthesizing the Pt-M core-shell structure for possibly superior ORR catalytic performance. The facile oxygen annealing treatment may thus pave a way for designing and tailoring the structure and performance of core-shell Pt-Co nanocatalysts.

## Methods

### Sample preparation

Carbon-supported Pt and Pt_3_Co powder catalysts (Pt/C and Pt_3_Co/C) with a metal loading of 40 wt% were obtained from Tanaka Kikinzoku Kogyo (TKK). Since Pt_3_Co bimetallic catalysts are typically prepared under high-temperature (800–1000 °C) carbothermal reduction of Pt and Co precursors in an inert atmosphere, both catalyst samples were aged at 900 °C in N_2_ for 1 h, then cooled rapidly, in order to make their initial states more comparable. The Pt_3_Co/C sample was first dispersed in solvent, and the suspension was deposited directly onto a thermal E-chip, which is equipped with a thin ceramic heating membrane controlled by the Protochips Atmosphere system. A second E-chip window was then placed on top of the thermal chip in the holder, creating a thin gas cavity sealed from the high vacuum of the TEM column. The Pt_3_Co nanoparticles were situated between two SiN membranes, each 30–50 nm in thickness, with a 5 micron gap in between (see Supplementary Fig. 2).

### Electron microscopy

Electron microscopy was performed on a JEOL JEM-3100-R05 transmission electron microscope equipped with two spherical aberration correctors and a 300 kV cold field emission gun. HAADF-STEM images were recorded using a convergence semi angle of 22 mrad, and inner- and outer collection angles of 83 and 165 mrad, respectively. To minimize beam irradiation, a relatively small beam current of 20 pA was used for imaging.

### First principle calculations

Calculations based on density functional theory (DFT) were carried out with the Vienna Ab-initio Simulation Package (VASP)^41–44^ along with the projector augmented wave (PAW) method^45, 46^. The exchange and correlation interactions among electrons were treated at the level of the spin-polarized generalized gradient approximation (GGA), using the Perdew-Burke-Ernzerhof (PBE) functional^47^. An energy cutoff of 600 eV was used for the plane-wave basis expansion. In order to mimic the possible surfaces for the ordered Pt_3_Co nanoparticle, we constructed every possible surface model within a reasonable unit cell, as shown in Supplementary Fig. 11, and added the vacuum gap of about 15.0 Å thick between the periodic slabs. During the geometry optimization, the middle three layers of all slabs were fixed, while all other layers were completely relaxed. A 7 × 7 × 1 Monkhorst-Pack k-point mesh^48^ was used to evaluate integrals in the reciprocal space. The criteria for structural optimization are as follows: (1) the atomic force on each atom is < 0.01 eV/Å and (2) the energy convergence is better than 10^−7^ eV.

### Data availability

The data that support the findings of this study are available from the corresponding author on reasonable request.

## Electronic supplementary material

Supplementary Information
