# Supplementary material for: In situ atomic-scale observation of oxygen-driven core-shell formation in Pt3Co nanoparticles
Source: Nat Commun. 2017 Aug 7;8:204. doi: 10.1038/s41467-017-00161-y (PMC5547122; doi:10.1038/s41467-017-00161-y)
Supplement: Supplementary file 1 — Supplementary Information [file 41467_2017_161_MOESM1_ESM.pdf]

File Name: Supplementary Information
Description: Supplementary Figures, Supplementary Tables, Supplementary Notes, Supplementary Methods and Supplementary References

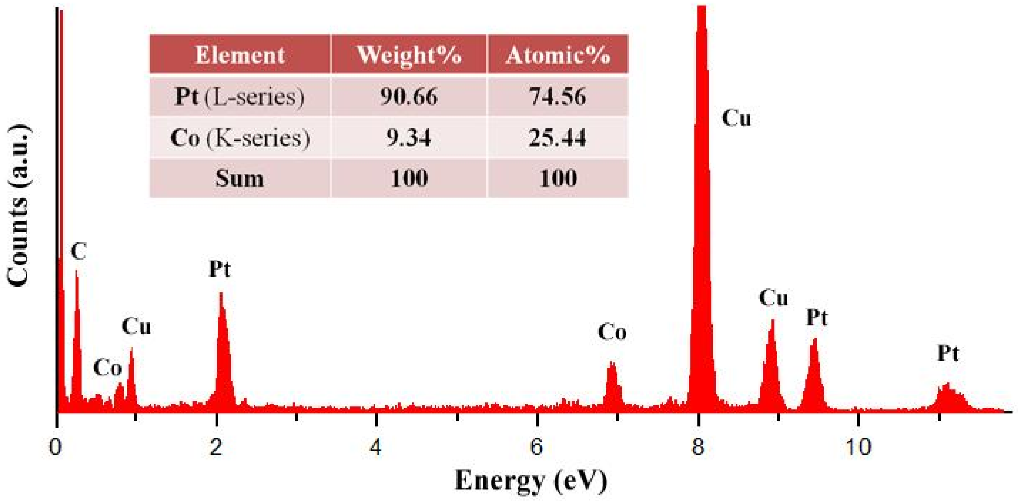

Supplementary Figure 1 | Energy-dispersive X-ray spectrum of the as-received Pt_3Co/C sample. The inset shows the corresponding elemental quantification, confirming the chemical composition of 75 at% Pt + 25 at% Co. The Cu peaks in the spectrum are generated from the copper grid.

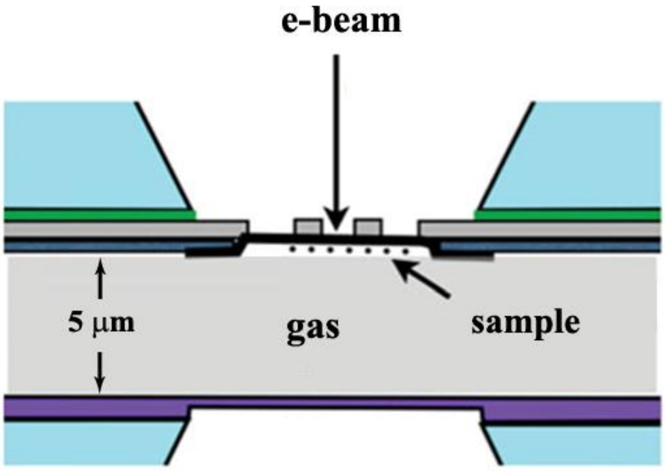

Supplementary Figure 2 | Schematic showing the cross section view of the assembled gas cell with catalyst powder loaded.

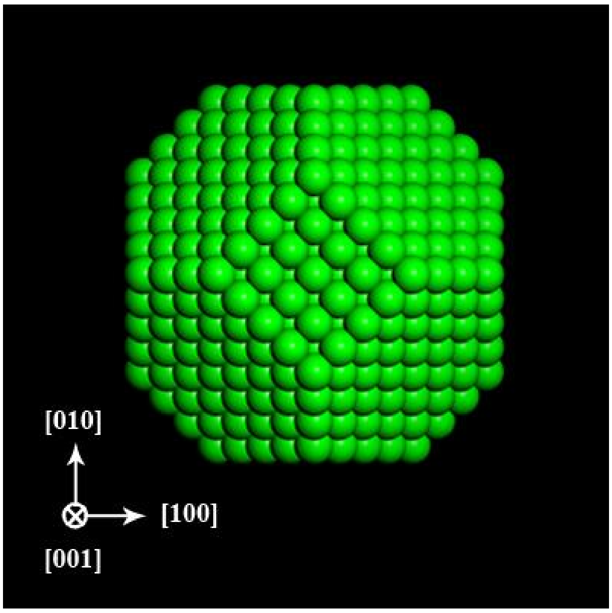

Supplementary Figure 3 | Projective model of a truncated octahedron Pt nanoparticle along the <001> zone axis.

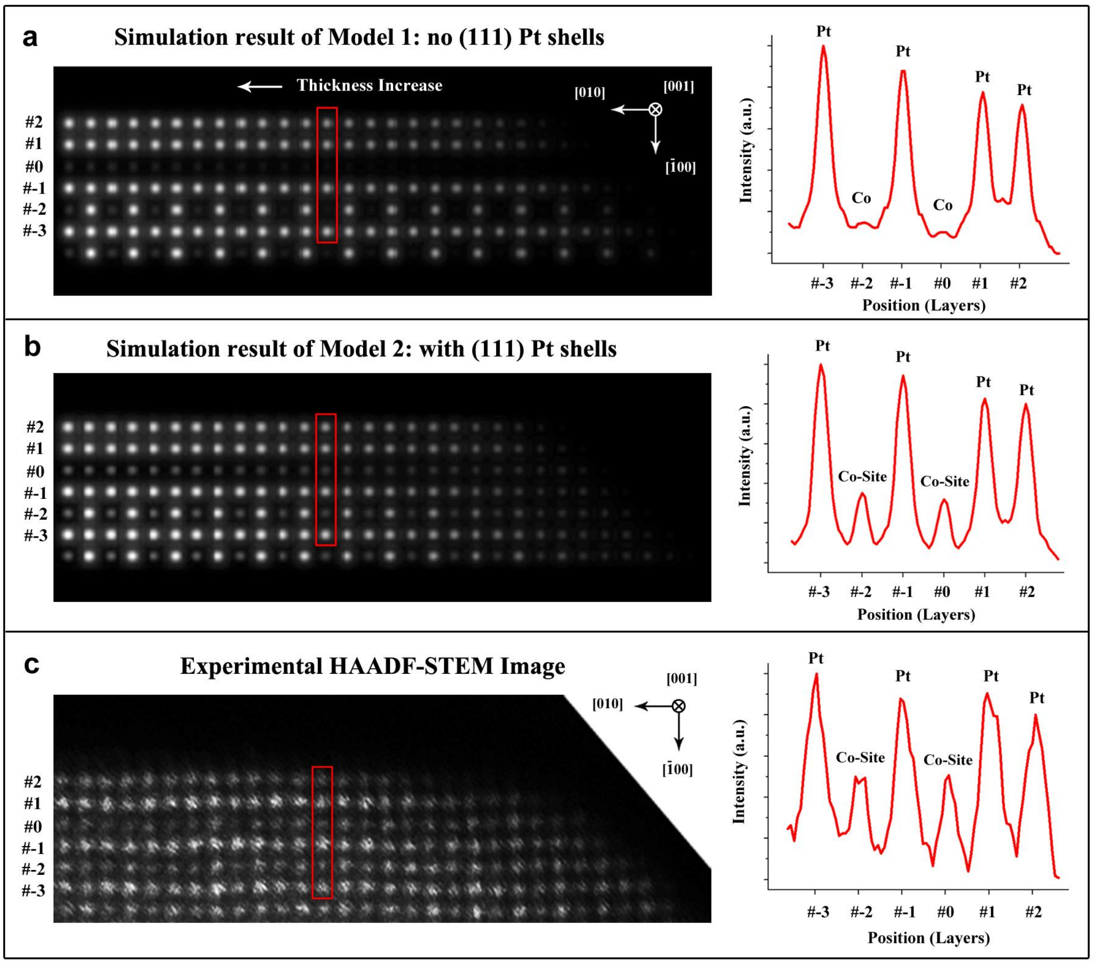

Supplementary Figure 4 | Comparison between the experimental and simulated HAADF-STEM images. a, Image simulation based on Model 1, which has no Pt shell on {111} surface. The intensity profile taken from a (020) Pt-Co mixed plane (indicated by red box) shows a very weak intensity at the Co columns. **b,** Image simulation based on Model 2, which has a two-layer segregated Pt shell on {111} surface. The intensity at the Co columns is enhanced by the Pt-shells on the (111) surfaces. **c,** Experimental HAADF-STEM image taken after 30 minutes oxygen annealing at 720 °C. The intensity profile taken from the same (020) plane shows the enhanced intensity at the Co-site columns, and matches the simulation result in **b**. (More details about the atomic models are provided in the Supplementary Note 1.)

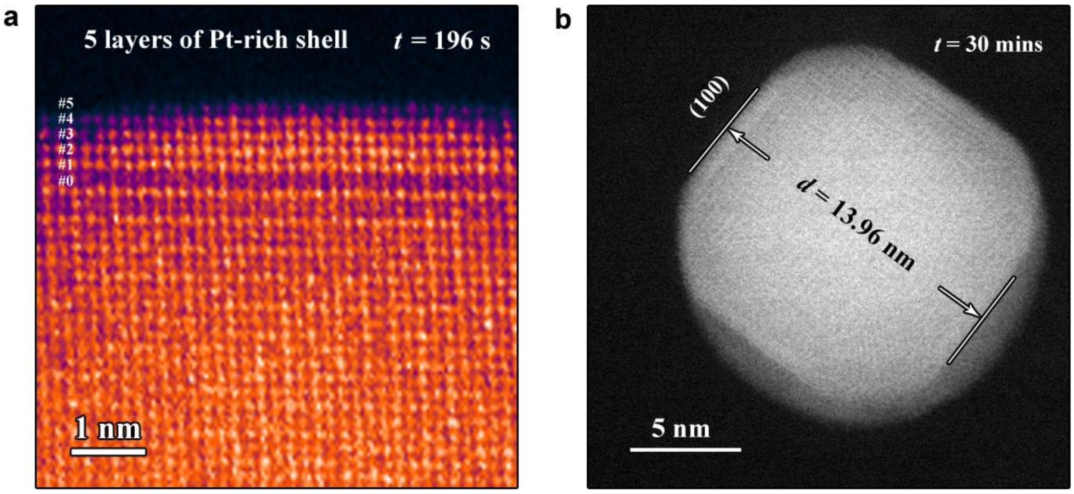

Supplementary Figure 5 | Final state of the Pt_3Co nanoparticle after oxygen-driven core-shell formation. a, Atomic resolution STEM image taken at 196 s showing the 5-layer Pt-rich shell on the (100) surface. **b,** Final state of the nanoparticle with the diameter of 13.96 nm.

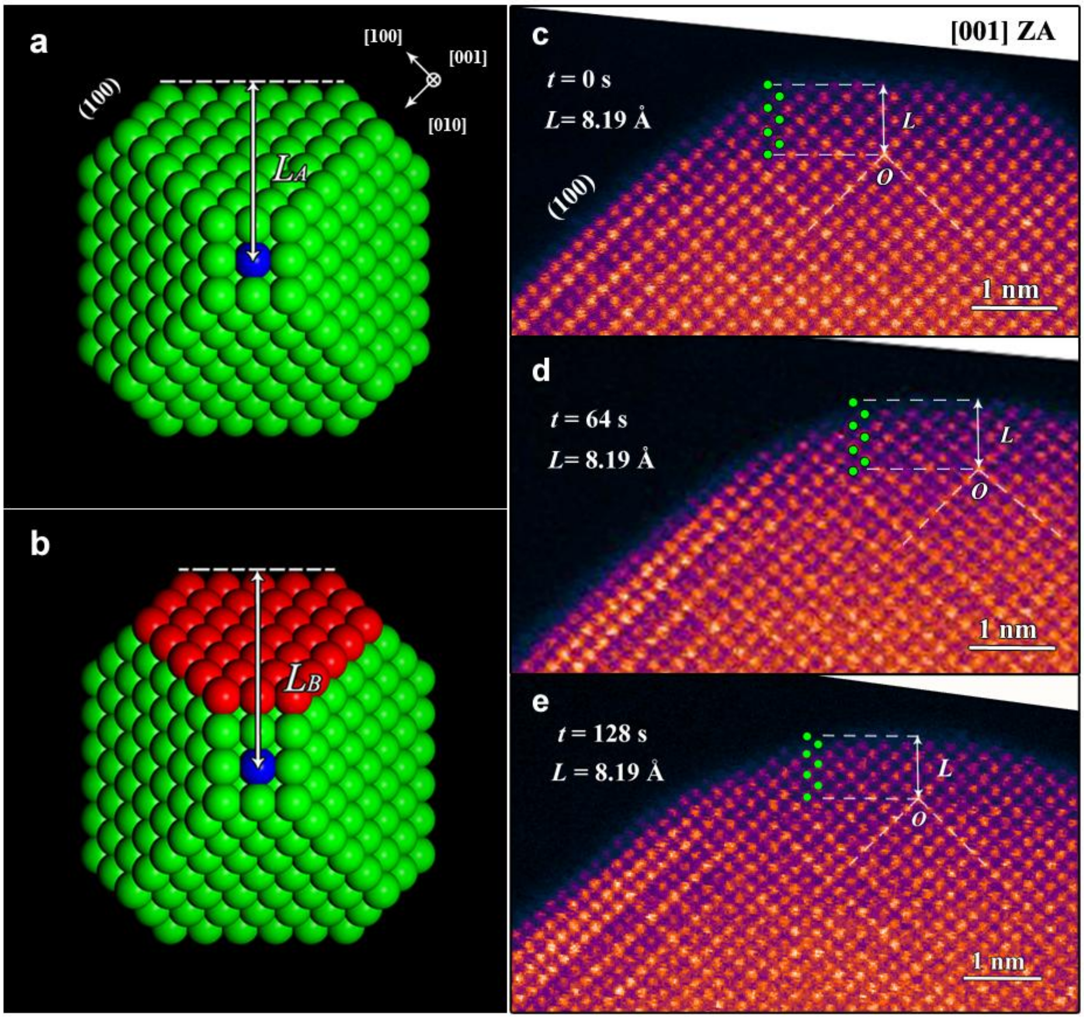

Supplementary Figure 6 | Measurements of *L* showing the status of (111) surface growth of a Pt_3Co nanoparticle. a, b, Truncated octahedron models before **a** and after (111) layer growth **b**. Green and red spheres represent the original atoms before growth and the newly grown atoms, respectively. The blue sphere is for the reference point. **c, d, e** Measurements of *L* in sequential STEM images taken during oxygen-driven Pt-shell formation.

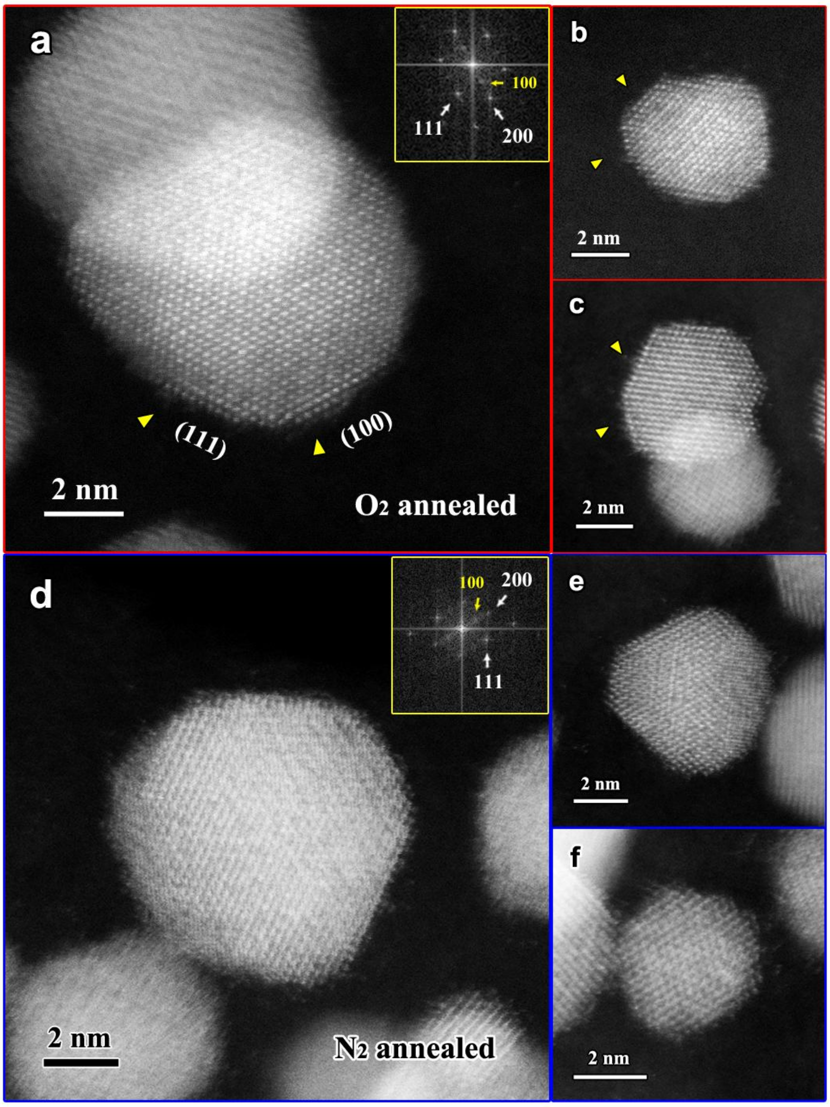

Supplementary Figure 7 | HAADF-STEM images of the *ex-situ* annealed Pt_3Co samples. a-c, Oxygen annealed Pt_3Co nanoparticles. The Pt-rich surface with brighter contrast is indicated by the yellow arrows. **d-f,** Nitrogen annealed Pt_3Co nanoparticles with no Pt enriched surface.

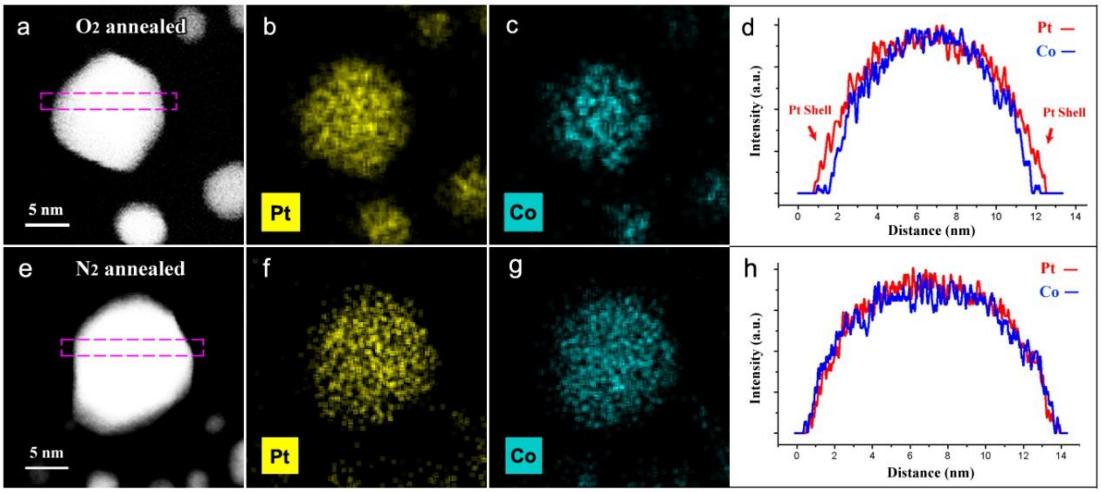

Supplementary Figure 8 | EDS elemental mapping of the *ex-situ* annealed Pt_3Co samples. a-d Results of *ex-situ* oxygen annealed Pt_3Co sample. Intensity profiles d are collected from the rectangular box in **a**. **e-h** Results of *ex-situ* nitrogen annealed sample. Intensity profiles h are collected from the rectangular box in **e**.

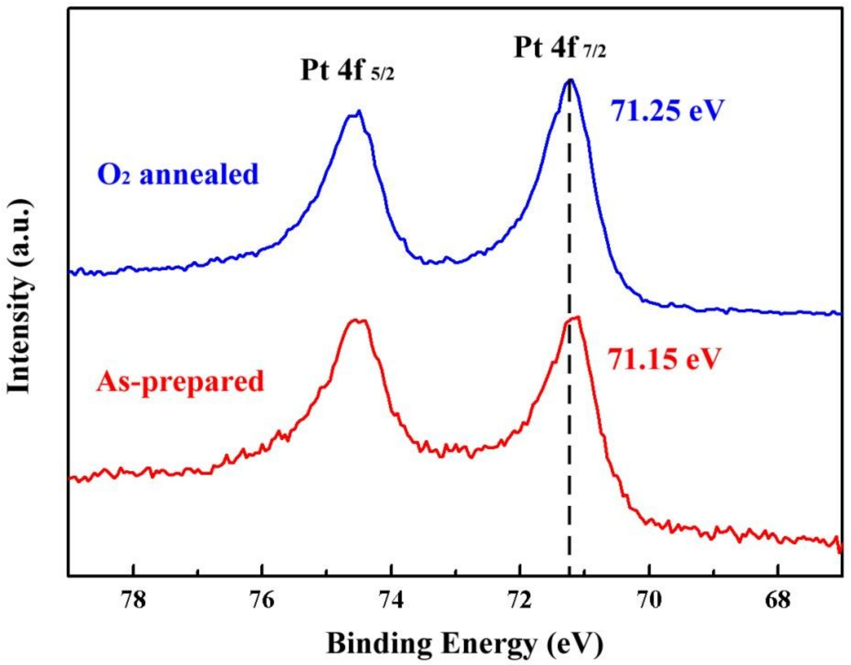

Supplementary Figure 9 | Normalized Pt 4f X-ray photoelectron spectra of oxygen annealed (blue) and as-prepared (red) Pt_3Co samples.

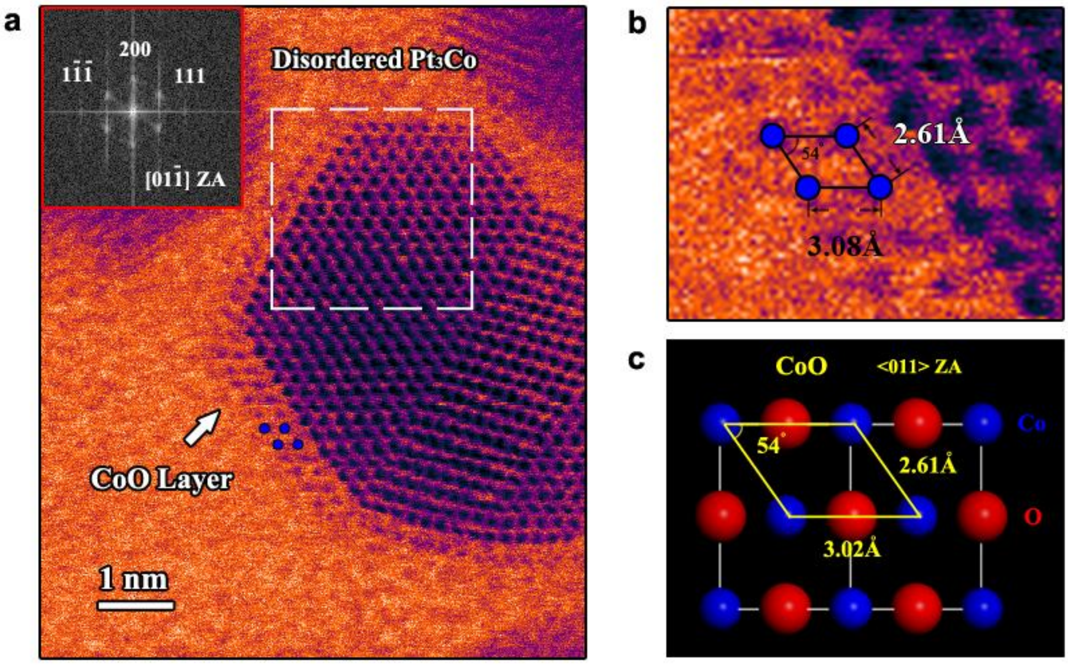

Supplementary Figure 10 | Oxidizing results of the disordered Pt_3Co nanoparticle. a, CoO layers is found in a disordered Pt_3Co particle after the oxygen annealing at 500 ºC for 30 minutes. Inset is the FFT pattern taken from the area indicated by the white box, demonstrating its disordered structure. **b,** Enlarged HRSTEM image illustrating the periodical unit cell of the oxide layer. **c,** Projection of CoO atomic model along <011> zone axis. Blue and red spheres represent the Co and O atoms, respectively.

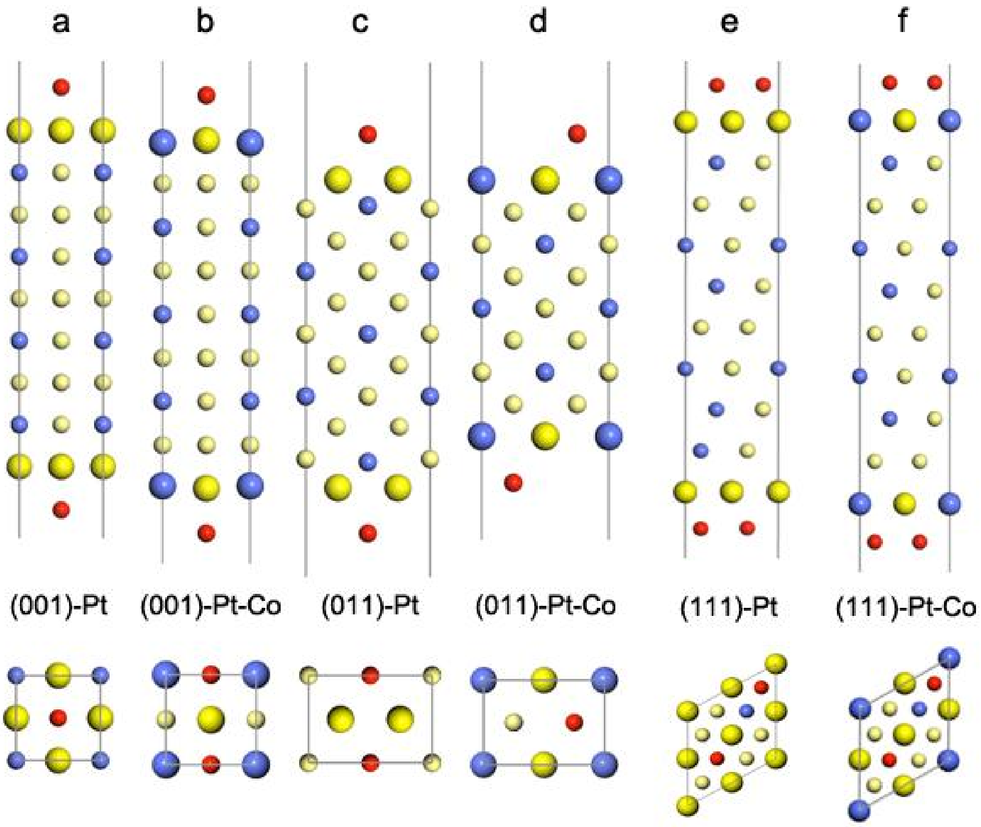

Supplementary Figure 11 | Schematic atomic geometries of oxygen atoms absorbed on **a, c, e,** pure Pt surfaces and **b, d, f,** Pt-Co mixed surfaces. The upper graphics are the side views, and the lower graphics are the corresponding top views for the (100), (110) and (111) facets. Yellow, blue and red spheres represent the Pt, Co, and absorbed O atoms, respectively.

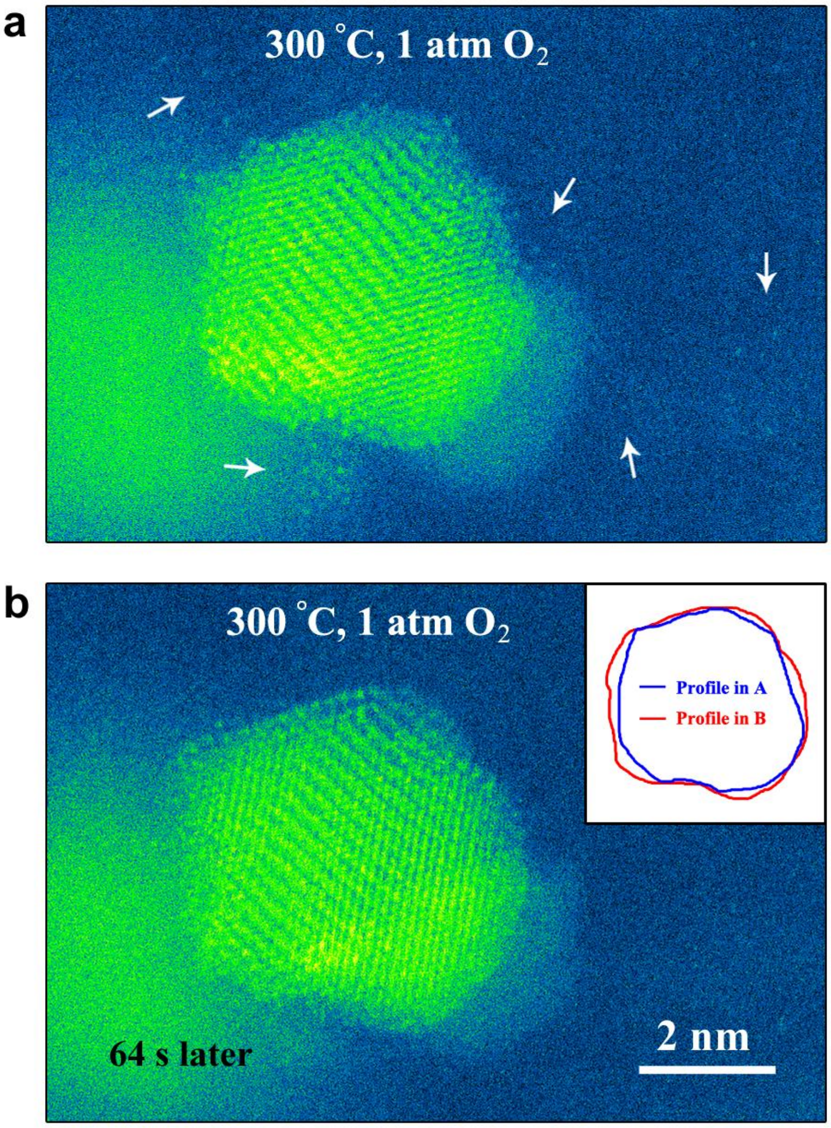

Supplementary Figure 12 | *In-situ* STEM images showing the high mobility of Pt atoms/clusters in the oxygen environment. a, Visible Pt atoms/clusters (indicated by white arrows) were attaching on the surface of the Pt_3Co nanoparticle. **b,** After an elapsed time of 64 s, the smooth surface was formed by the Pt atoms' diffusion. Inset shows the comparison of the particle profile in **a** and **b**, indicating the volume of the particle was increasing due to the Pt atoms' diffusion.

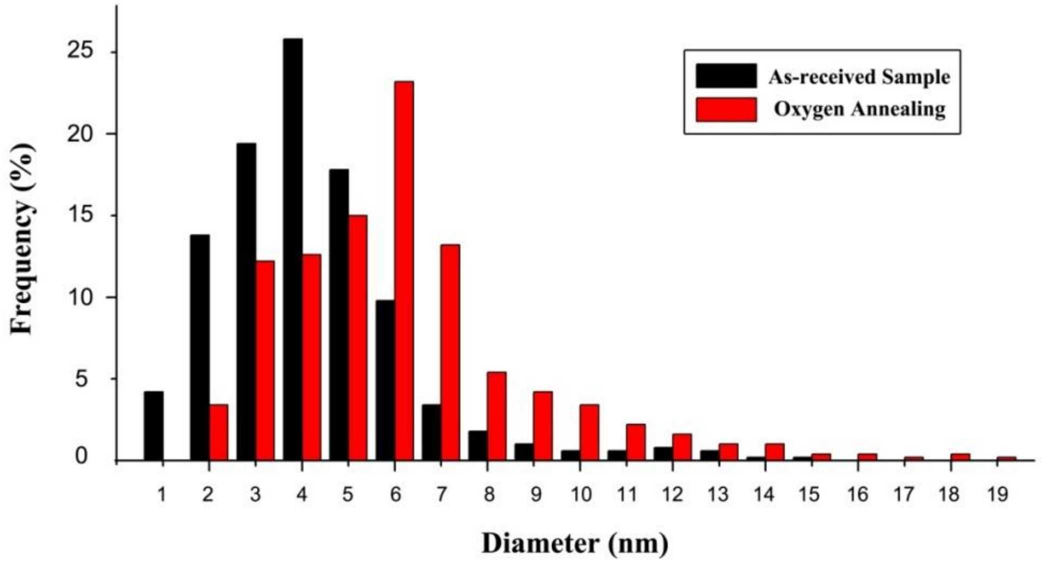

Supplementary Figure 13 | Pt_3Co particle size distributions of sample before (black) and after 30-minute oxygen annealing (red).

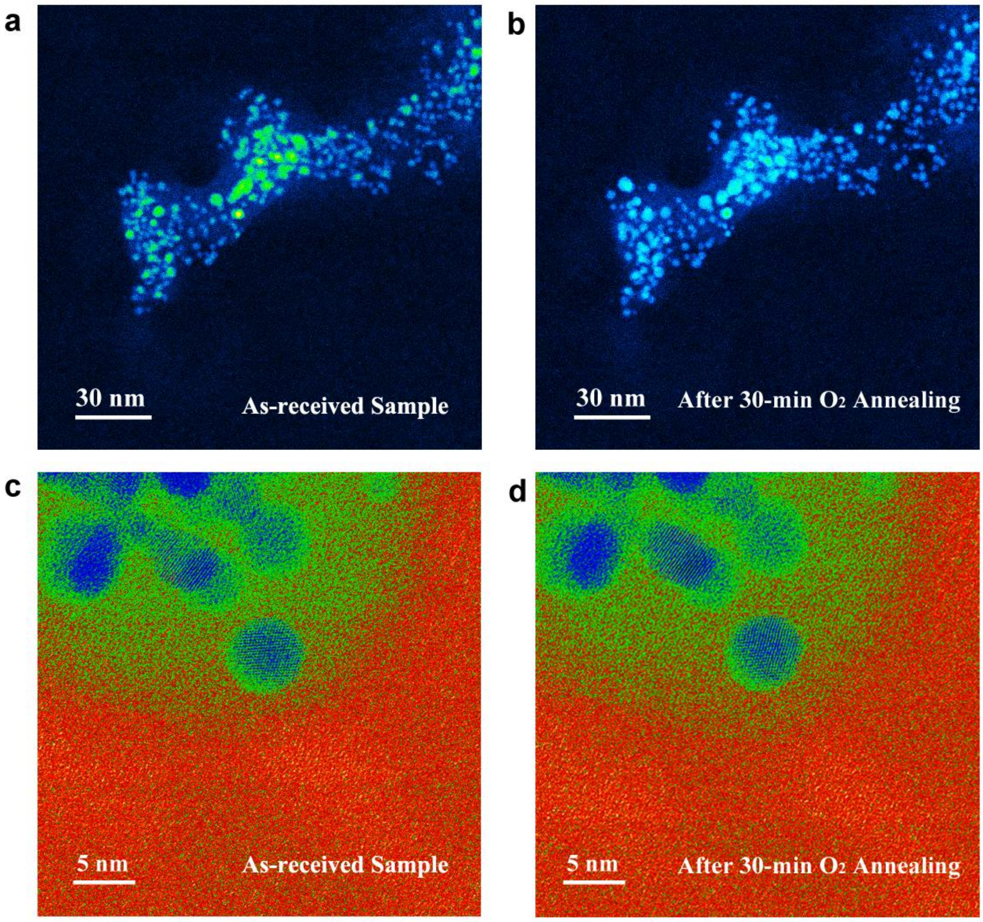

Supplementary Figure 14 | Comparison of the Pt_3Co/C sample before and after 30-minute oxygen annealing showing the stability of the carbon support. a, b are the low magnification comparison showing an overall view of the carbon support (the light blue area besides the nanoparticles) . **c, d** are the high magnification images demonstrating no obvious loss in the carbon support (the green area).

Supplementary Table 1 | XPS results showing the surface Pt/Co ratios of the as-prepared and oxygen annealed Pt_3Co samples.

Sample	Surface Pt/Co ratio
As-prepared Pt_3Co	2.88
Oxygen-annealed Pt_3Co	4.81

Supplementary Table 2 | Statistics for particle sizes of as-received and oxygen-annealed Pt_3Co nanoparticles.

Sample	Mean Size (nm)	Minimum (nm)	Maximum (nm)
As-received Pt_3Co	4.81	1.49	15.60
Oxygen-annealed Pt_3Co	6.63	2.21	19.21

Supplementary Note 1 | Construction of structural models of Pt_3Co nanoparticles

Model 0: A basic ordered Pt_3Co model with no core-shell structure. An ordered intermetallic Pt_3Co nanoparticle with no core-shell structure was first established. The diameter across two opposite (100) surfaces was 12.75 nm, the same dimension as the particle we observed in the experiment. The morphology of the particle was a truncated octahedron consisting predominantly of {100} and {111} facets.

Model 1: An ordered Pt_3Co model with Pt segregation only on {100} surfaces. For comparison, we only modify the {100} surfaces of Model 0: Model 1 thus only has Pt segregation on {100} surfaces but not on {111} surfaces.

Model 2: An ordered Pt_3Co model with Pt segregation on all the surfaces. This model was constructed from Model 0 by setting the top three layers on all {100} and {111} surfaces to a Pt-Pt-Co configuration.

Model 3: An ordered core-shell Pt_3Co model with additional Pt layers on {100} surfaces. This model describes the extra Pt layers that grow on {100} surfaces of the Pt segregated Pt_3Co nanoparticle. To construct this model, extra {100} Pt layers were added onto Model 2.

(xyz structure files of the models are available upon request to S. D.)

Supplementary Note 2 | Final state of the Pt_3Co nanoparticle during oxygen-driven core-shell formation

In the manuscript, Fig. 3 shows the continuous layer-by-layer Pt shell growth on (100) surfaces of a Pt_3Co nanoparticle. At the time t=128 s, a 4-layer Pt-shell was formed and the measured diameter, d, was 13.57 nm.

Supplementary Fig. 5 exhibits the final state of this nanoparticle during oxygen-driven core-shell formation. Supplementary Fig. 5a, the high resolution STEM image taken at the time t=196 s, shows the 5-layer Pt shell on the (100) surface. The diameter at this time was measured to be 13.96 nm.

In the following time, the Pt shell stopped growth after the #5 Pt layer was formed. As shown in Supplementary Fig. 5b, the diameter, d, was still 13.96 nm in the next 30 minutes, and it remained unchanged upon further annealing in oxygen at 300 ºC.

Supplementary Note 3 | Analysis of (111) surfaces of Pt_3Co nanoparticles during oxygen annealing

The layer-by-layer growth on (100) surfaces was identified by direct observation along the [001] zone axis (Figs. 2, 3 in the manuscript). Although (111) facets are not parallel to the [001] zone axis, further analysis is able to reveal the status of (111)

surfaces. The following analysis will be based on the geometry of a truncated octahedron. Therefore it is not necessary to distinguish Pt atoms and Co atoms in the figures and models.

Supplementary Fig. 6a is a truncated octahedron model projected along the [001] zone axis. Green spheres represent either Pt atoms or Co atoms for simplicity. For comparison, Supplementary Fig. 6b shows an extra atom layer (the red one) growing on a certain (111) surface. As illustrated in Supplementary Fig. 6, the length, L, along the [110] direction is defined as the projected distance from a reference point to the edge of the (111) surface. In Supplementary Figs. 6a and 6b, we set the octahedron center as the reference point (indicated by the blue sphere). Then it should been found that the measured length, L_B, (in Supplementary Fig. 6b) will be longer than L_A (in Supplementary Fig. 6a) due to the growth of an extra (111) layer.

Therefore, the status of (111) surface can be reflected in the length, L, while observing along the [001] zone axis. Similarly, if the top layer of a (111) surface has diffused away, the measured length, L, would become shorter. Increase/decrease of length, L, thus corresponds to the growth/loss of a (111) surface.

Supplementary Fig. 6c (t=0 s), 6d (t=64 s), and 6e (t=128 s) are enlarged false color panels corresponding to Figs. 3a, 3b, and 3c in the manuscript, showing the status of a (111) surface during the oxygen-driven core-shell formation process. Here, we set an obvious feature, the intersection point of two high-contrast atom layers (see point O in Supplementary Fig. 6c) as the reference point and then measure the projected distance (L) from point O to the edge of a (111) surface.

According to the measurements, it is clear that the distances L_{0s}, L_{64s} and L_{128s} are all the same length, 8.19 Å, corresponding to the projected distance between three (110) layers. No obvious structure change could be found in these high-resolution images. This result indicates that no growth or diffusion took place on the (111) top surface during the time period while a Pt shell was growing on the (100) surfaces.

Supplementary Note 4 | *Ex-situ* results of the oxygen annealed Pt_3Co nanoparticles

Ex-situ oxygen annealing was performed on the original Pt_3Co/C sample in a furnace tube, while nitrogen annealing was also carried out for comparison. All the conditions (temperature, heating rate and annealing time) were similar to those used in the *in-situ* experiment. Either the oxygen or the nitrogen annealing was first performed for 30 minutes under 720 °C (at a very high heating rate) and then for an additional 15 minutes under 300 °C.

Aberration-corrected scanning transmission electron microscopy (AC-STEM) was performed for the characterization of the *ex-situ* annealed samples by using a JEM-ARM300F Grand ARM transmission electron microscope. Here, Supplementary Figs 7a-c present the typical high-angle annular dark field (HAADF) images of the

oxygen annealed Pt_3Co nanoparticles. As shown in Supplementary Fig. 7a, the alternating (200) planes of bright-dark contrast corresponding to the ordered intermetallic Pt_3Co structure can be observed. The fast Fourier transform (FFT) pattern clearly illustrates the (100) superlattice spots, which characterized the intermetallic $L1_2$ phase. Meanwhile, it is clear that (100) and (111) surface region exhibits a brighter contrast in the HAADF image, demonstrating the structure of the Pt-rich shell on the ordered Pt_3Co core. In addition, more examples in Supplementary Figs. 7b and 7c also show the ordered core-shell structure of the Pt_3Co nanoparticles through the *ex-situ* O_2 annealing treatment.

For comparison, Supplementary Figs. 7d-7f present the HAADF images of the N_2 annealed Pt_3Co sample. In Supplementary Fig. 7d, the alternating (200) planes of bright-dark contrast can also be found, indicating the transition from disordered to ordered structure under the high temperature of 720 ºC. However, as shown in Supplementary Fig. 7d, the surface region of the Pt_3Co nanoparticle does not show a brighter contrast. Similarly, this point is also demonstrated in other N_2 annealed Pt_3Co particles (Supplementary Figs. 7e and 7f, which show that the surface is not enriched with Pt).

Furthermore, energy dispersive X-ray spectroscopy (EDS) was also performed on these two *ex-situ* annealed samples. Supplementary Fig. 8 is the EDS element maps of Pt_3Co nanoparticles, which were treated by O_2 and N_2 annealing, respectively. Results from the oxygen annealed sample (Supplementary Figs. 8a-8d) reveal Pt surface enrichment since the profile of the Pt map is bigger than that of the Co map. Intensity profiles, collected from the rectangular box in Supplementary Fig. 10a and aligned to the maximum value of Pt and Co intensities clearly reveal the Pt segregation at the surface region of the oxygen annealed Pt_3Co nanoparticles. However, in contrast, Pt surface enrichment is not found in the N_2 annealed sample (Supplementary Figs. 8e-8h). It is obvious that the profiles of the elemental maps of Pt and Co are almost the same in Supplementary Figs. 8f and 8g. The homogeneous distribution is confirmed in the intensity profiles, which were from the rectangular box in Supplementary Fig. 10e, and no Pt segregation is observed at the surface region of the nanoparticle.

Therefore, these *ex-situ* results demonstrate the oxygen-driven effect on the ordered core-shell formation of Pt_3Co nanoparticles. Meanwhile, the results also confirm that our *in-situ* findings also apply under similar *ex-situ* conditions, ruling out the electron beam effect in the *in-situ* experiment.

Supplementary Note 5 | XPS analysis of the oxygen annealed Pt_3Co nanoparticles

X-ray photoelectron spectroscopy (XPS) was performed on the *ex-situ* oxygen annealed Pt_3Co sample and the as-prepared one. The experiment was carried out on a Kratos Analytical AXIS Supra spectrometer utilizing monochromatic Al Kα radiation

(1486.7 eV, 250 W) under ultra-high vacuum conditions (~10^{-9} Torr). The electron inelastic mean free path (λ) is about 2.0 nm, and the highest signal contribution originates from the topmost surface region (65% of the signal comes from first 2.0 nm).

The Pt/Co ratios at the surface region are calculated from the corresponding Co 2p and the Pt 4f peak areas, where the Shirley background were subtracted, by the equation

$$\frac{X_{Pt}}{X_{Co}} = \frac{I_{Pt} / \sigma_{Pt}}{I_{Co} / \sigma_{Co}}$$

where X_i is the molar fraction, I_i is the integrated area of the XPS peaks, and σ_i is the photoelectron cross-section (Scofield factor) for the element *i* (*i*=Pt, Co).

The calculated Pt/Co ratios are presented in Supplementary Table 1. The surface Pt/Co ratio of the as-prepared sample (2.88) is very similar to the bulk value (3.0). In contrast, the sample treated by oxygen annealing shows a Pt/Co ratio of 4.81, indicating a Pt-rich composition at the surface region.

Moreover, Supplementary Fig. 9 shows the normalized Pt 4f photoelectron spectra of these two kinds of Pt_3Co samples. The binding energies were referred to the Au $4f_{7/2}$ signal at 84.0 eV from the sample holder, which was in electrical contact with the samples. In the low energy band (Pt $4f_{7/2}$), a small positive shift is observed in the Pt binding energies of O_2 annealed sample (71.25 eV) compared to that of the as-prepared Pt_3Co sample (71.15 eV). According to a theoretical explanation[1], this change in electronic structure properties is due to the variation in the surface atomic distribution of Pt in the Pt_3Co nanoparticles. Since a strain is introduced on the Pt shell due to a smaller lattice parameter of the Pt_3Co core, the shell Pt atoms are squeezed closer, leading to the increased overlap of the d-orbitals, and consequently the band broadens. Then the center of the d-band moves to low energy in order to maintain the same filling degree[2]. As the density of states at the Fermi level decreases, it is increasingly more difficult to "ionize" the metal, resulting in the increase of Pt XPS binding energies[3]. Such a phenomenon is also found in the published literature relating to the Pt-Co core-shell structure[4]. Particularly, Zhang et al.[5] also observed a similar positive shift (0.1 eV) of Pt $4f_{7/2}$ binding energy when the surface of Pt_3Co nanoparticles was enriched with Pt through an annealing treatment.

Supplementary Note 6 | Size distribution of Pt_3Co nanoparticles before and after oxygen annealing

Size distributions of at least 500 Pt_3Co nanoparticles were determined by *in-situ* diameter measurements. Supplementary Fig. 3 shows the size histograms before (black) and after oxygen annealing (red).

Statistics for particle sizes are listed in Supplementary Table 2.The diameter of Pt_3Co nanoparticles before annealing ranged from 1.49 nm to 15.60 nm, and the mean

size was about 4.81 nm. After *in-situ* annealing for 30 minutes (under 760 Torr oxygen, 720 ºC), the diameter was found to range from 2.21 nm to 19.21 nm, and the mean size was about 6.63 nm. These results show that this annealing treatment in an oxygen atmosphere did not induce severe sintering or coalescence of Pt_3Co nanoparticles.

Supplementary Note 7 | Consideration of the effect of oxygen annealing on the amorphous carbon support of the Pt_3Co/C sample

Considering the fact that bulk carbon can burn and may be converted into gas (CO or CO_2) upon sample heating within the oxygen environment, it is necessary to both (1) examine the possible effect of this reaction on gas composition and (2) directly assess the stability of the carbon support.

First, we estimate the moles of carbon and oxygen in the cell under our experimental condition. Supplementary Fig. 2 is a schematic diagram showing the cross section view of the Protochips Atmosphere™ gas cell. The dimensions of the cell are about 3cm×1cm×5μm. The area for sample loading is 100 μm×100 μm, and the gap between the chip and the window is about 5 μm.

If we assume that the in-cell gas can be treated as an ideal gas[6], following the ideal gas law,

$$pV = nRT \tag{1}$$

where p is the gas pressure, V is the gas volume, n is the number of moles, R is the universal gas constant 0.08206 (atm·L)/(mol·K), and T is the temperature, the number of oxygen moles (n_O) in the gas cell under our experimental condition (V=1.5×10^{-6} L, p=1.0 atm, T=993 K) is

$$n_O = \frac{pV}{RT} = \frac{1\times1.5\times10^{-6}}{0.08206\times993} = 1.8\times10^{-8}\ \text{mol} \tag{2}$$

The typical volume of catalyst sample is less than

$$V_{\text{sample}} = 10\mu\text{m}\times10\mu\text{m}\times1\mu\text{m} = 1\times10^{-16}\text{m}^3 \tag{3}$$

Assuming the sample is all amorphous carbon, then the carbon moles of the support (n_C) is

$$n_C = \frac{\rho_C \cdot V_{\text{sample}}}{M_C} = \frac{2\times10^3\times1\times10^{-16}}{12\times10^{-3}} = 1.7\times10^{-11}\ \text{mol} \tag{4}$$

where ρ_C is the mass density of amorphous carbon (2.0 g/cm^3), and M_C is the molar mass of carbon (12 g/mol).

This means the oxygen moles always far exceeds the carbon moles in the cell under our experimental condition. Even if the carbon support can react fully with the oxygen gas, the carbon oxide (CO or CO_2) produced is only of order 0.1% of all the gas in the cell. It can thus be ensured that the gas composition in the cell is oxygen with purity over 99.9% during the experiment.

However, by direct observation of the carbon support, we can demonstrate its stability during the annealing experiment. Supplementary Fig. 14a, b are the low magnification STEM images showing the Pt_3Co/C sample before and after the oxygen annealing process, respectively. False color images are used to enhance the contrast of the carbon support in the gas cell. The light blue part besides the nanoparticles is the carbon support. From these two images, it can be seen that the profile of the carbon support was essentially unchanged after the oxygen annealing, while some of the nanoparticles exhibited different contrast due to the tilting of their orientations.

Supplementary Fig. 14c, d show the detailed characterization of the carbon support before and after the oxygen annealing, respectively. The green area represents the carbon support and the blue particles are the Pt_3Co catalyst. Red area here represents the background (the cell window) on which some tiny and separated green spots should be the noise. Although some of the particles exhibit a slight degree of sintering after 30-minute oxygen annealing, the carbon support shows no noticeable change, according to these two images. Therefore, no obvious loss of the amorphous carbon can be found according to the *in-situ* observation. The carbon support of the Pt_3Co/C catalyst is thus quite stable during the 30-minute static oxygen annealing (under 760 Torr oxygen, 720 ºC).

Overall, we conclude that the carbon support is stable during the 30-minute oxygen annealing, and its possible combustion would not, in any case, significantly affect the oxygen environment inside the gas cell.

Supplementary Methods

HAADF-STEM image simulation

After the atomic models (see Supplementary Note 1) were established, a (100) surface slab (11.59 nm × 11.59 nm × 1.37 nm) was extracted from each model for the image simulation.

HAADF-STEM image simulation was performed using the QSTEM simulation package[7]. The simulation was carried out using a 512×512 pixel area and a single slice thickness of 1.93 Å. The microscopy parameters used for the simulations were the same as those for imaging.

Supplementary References

1. Weinert, M. & Watson, R. E. Core-level shifts in bulk alloys and surface adlayers. *Phys. Rev. B* **51**, 17168 (1995).

2. Wakisaka, M. *et al.* Electronic structures of Pt-Co and Pt-Ru Alloys for CO-tolerant anode catalysts in polymer electrolyte fuel cells studied by EC-XPS. *J. Phys. Chem. B* **110**, 23489 (2006).

3. Vorokhta, M. *et al.* Surface composition of magnetron sputtered Pt-Co thin film catalyst for proton exchange membrane fuel cells. *Appl. Surf. Sci.* **365**, 245–251 (2016).

4. Zhang, X. *et al.* Strain effect of core-shell Co@Pt/C nanoparticle catalyst with enhanced electrocatalytic activity for methanol oxidation. *J. Electrochem. Soc.* **159**, B270-B276 (2012).

5. Zhang, Z. C. *et al.* Engineering phase and surface composition of Pt_3Co nanocatalysts: A strategy for enhancing CO tolerance. *Nano Energy* **34,** 224-232 (2017).

6. Moran, M. J. & Shapiro, H. N. *Fundamentals of Engineering Thermodynamics.* John Wiley & Sons, Inc., Hoboken, NJ, 2000.

7. Koch, C. T. *Determination of Core Structure Periodicity and Point Defect Density along Dislocations*. Arizona State University, Phoenix, AZ, 2002
